# Post-myocardial Infarction Left Ventricular Aneurysm With Contained Rupture and Hemopericardium

**DOI:** 10.7759/cureus.56506

**Published:** 2024-03-19

**Authors:** Moiz Saeed, Rand Sabanci, Adolfo Martinez, Andrew G Kim, Rohan M Prasad, Christopher Hanson, Michael Kehdi

**Affiliations:** 1 Internal Medicine, Michigan State University, East Lansing, USA; 2 Cardiology, Sparrow Hospital Thoracic and Cardiovascular Institute, Sparrow Hospital, Lansing, USA

**Keywords:** cardiac tamponade, hemopericardium, pericardial effusion, acute pericardial effusion, ventricular aneurysm, lva, complication of myocardial infarction, myocardial infarction, echocardiography

## Abstract

Left ventricular aneurysms (LVAs) represent a rare yet critical complication arising from late-presenting myocardial infarction (MI). Here, we present the case of an 88-year-old male with chest pressure, elevated troponin, B-type natriuretic peptide, and lactate. The electrocardiogram showed sinus tachycardia and an old right bundle branch block. The patient was started on heparin infusion, but progressively worsening hypotension necessitated transfer to the intensive care unit and the initiation of vasopressors. The echocardiogram identified a focal aneurysm in the mid-anterolateral wall, moderate pericardial effusion with a coagulum, and tamponade physiology. Computed tomography angiography of the chest confirmed a moderate pericardial effusion with density consistent with hemopericardium. LVAs pose a substantial threat of cardiovascular morbidity and mortality. While echocardiography serves as the initial assessment method, supplemental imaging modalities may need to be utilized. Various complications have been reported with LVA, including thromboembolization, ventricular arrhythmias, pericardial effusion with tamponade, and left ventricular rupture which accounts for 5%-24% of all in-hospital deaths related to MI. Although LVAs are the most common mechanical complications following an MI, instances of contained aneurysm rupture leading to hemopericardium are infrequent and scarcely reported. High clinical suspicion and prompt imaging with echocardiography are essential for diagnosis. Determining the optimal timing and selection between surgical and percutaneous interventions necessitates additional research for informed decision-making.

## Introduction

Left ventricular aneurysms (LVAs) are a rare but well-documented complication of late presentation myocardial infarction (MI) [1,2]. Risk factors for LVA after MI include anterior wall infarction, delayed or incomplete reperfusion, and the absence of extensive collateralization. The complications associated with an LVA can be serious, encompassing conditions such as arrhythmias, congestive heart failure, and even rupture leading to tamponade [1,3]. Although left ventricular free wall rupture is a rare complication of MI (occurring in fewer than 1% of cases), it is noteworthy for its high mortality rate [4]. Early diagnosis and immediate surgical intervention are crucial, as the mortality rate is high in the absence of intervention. In this report, we present a case of an LVA with contained rupture and hemopericardium following a late presentation MI.

## Case presentation

**Figure 1:**
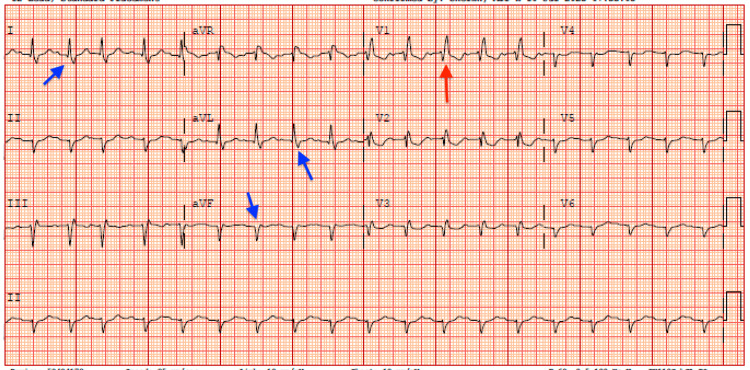
Electrocardiogram showing sinus tachycardia, old right bundle branch block as evidenced by the red arrow showing QRS prolongation and rSR pattern, left axis deviation as evidenced by blue arrows.

An 88-year-old male with a past medical history of hypertension, hyperlipidemia, and chronic obstructive pulmonary disease presented to the emergency department due to progressively worsening chest pressure that had been ongoing for two months before admission. Upon evaluation, he exhibited tachycardia, tachypnea, and normal blood pressure. His laboratory workup was concerning for elevated troponin levels in the 8,000s, an elevated B-type natriuretic peptide, acute kidney injury, and a lactate of 2.1 (Table 1). His electrocardiogram (EKG) displayed sinus tachycardia with no discernable ST-segment or T-wave changes (Figure 1). He was started on a heparin infusion and cardiology was consulted.

**Table 1:** Initial laboratory investigations.

Parameter	Value	Reference range
Troponin	8,883 ng/L	0-18 ng/L
B-type natriuretic peptide	212 pg/mL	0-100 pg/mL
Creatinine	1.5 mg/dL	0.60-1.40 mg/dL

**Figure 2:**
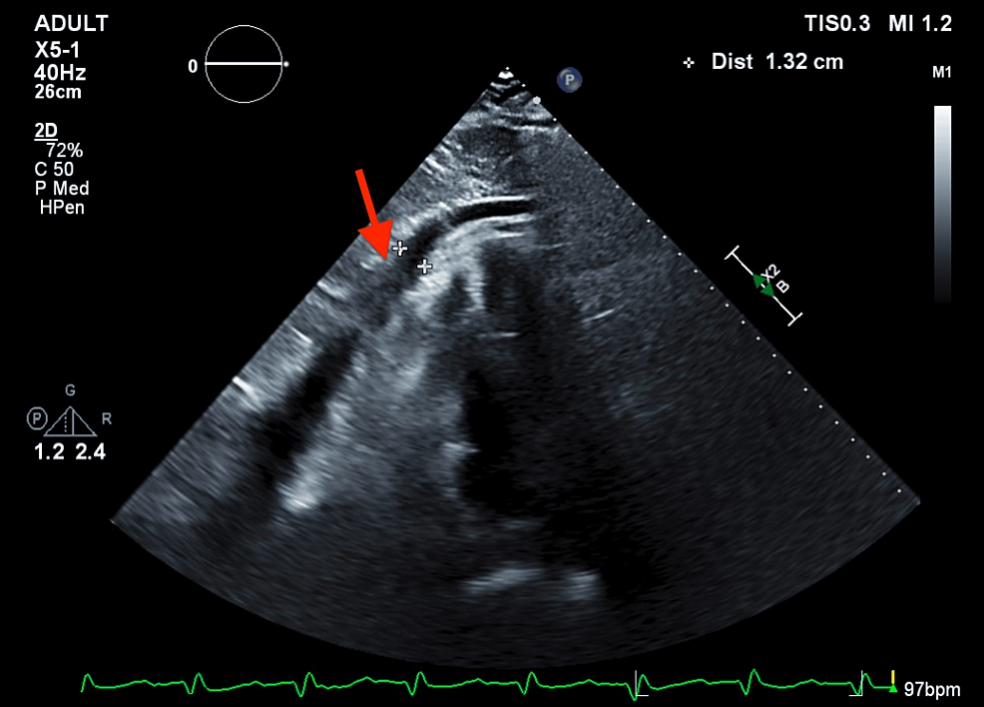
Echocardiogram four-chamber view with the red arrow pointing toward coagulum in pericardial effusion.

**Figure 3:**
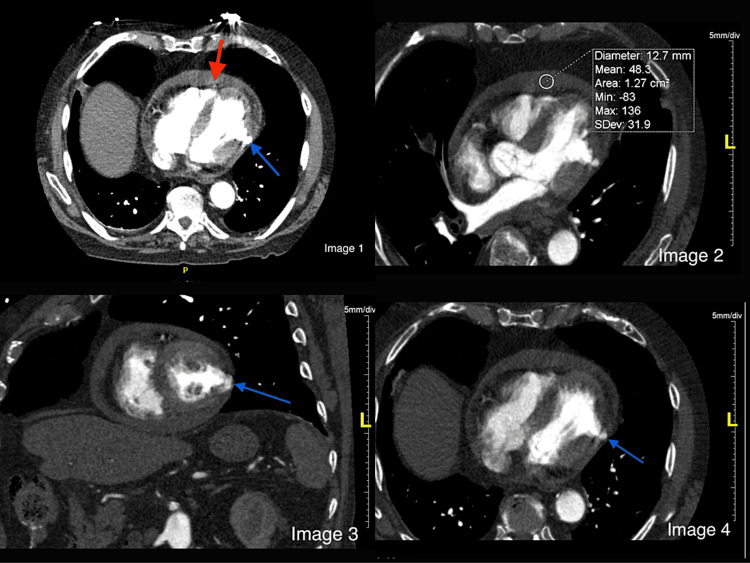
Image 1: CT chest axial section with the red arrow pointing to pericardial effusion and the blue arrow pointing toward a left ventricular aneurysm and potential rupture site. Image 2: CT chest three-chamber view with circle area of interest (AOI) revealing a mean fluid density of 48.3 Hounsfield units. Image 3: CT chest short-axis view of the heart with the blue arrow pointing toward a left ventricular aneurysm rupture site. Image 4: CT chest with the blue arrow pointing to left ventricular aneurysm rupture.

The following morning after admission, the patient reported worsening chest pain and became hypotensive. Despite adequate fluid resuscitation, there was no improvement, necessitating the initiation of vasopressors. Consequently, he was admitted to the intensive care unit for cardiogenic shock. An echocardiogram identified a focal aneurysmal area in the mid-anterolateral wall and moderate pericardial effusion with a coagulum (Video 1, Figure 2). Additionally, tamponade physiology with systolic right atrial collapse was observed. A computed tomography angiogram of the chest confirmed the presence of a moderate pericardial effusion and a left ventricular aneurysm with signs of possible rupture. Furthermore, the density of this pericardial fluid fell within the range typical of blood (greater than 30-45 Hounsfield units), strongly suggesting that the effusion consisted predominantly of blood (Figure 3).

**Video 1:** Echocardiogram four-chamber view significant for left ventricular aneurysm, moderate pericardial effusion, and coagulum.

Considering the patient’s high-risk status and suspicion of a late-presenting MI with contained free wall rupture, a shared decision-making process with the patient led to the decision not to proceed with a left heart catheterization. The heparin drip was discontinued due to concerns about the pericardial effusion possibly being hemopericardium. Subsequently, the patient was started on high doses of aspirin and colchicine to address pericarditis. A repeat echocardiogram was obtained due to worsening hemodynamic status that revealed consistent findings with the previous one, showing no increase in the size of the aneurysm or pericardial effusion. An echodensity, believed to be a thrombus, was noted at the apex outside the heart, causing external compression. Despite medical intervention, the patient’s condition continued to deteriorate until he ultimately passed away.

## Discussion

In patients experiencing acute MI, the presence of LVA is typically identified through imaging procedures performed during the diagnosis and management of the acute MI itself. Typically, the initial evaluation begins with echocardiography, as it is readily available. However, in cases where echocardiography does not provide a conclusive diagnosis or when further characterization of the ventricular wall is needed (such as distinguishing between an aneurysm and a pseudoaneurysm), additional imaging modalities can be utilized. Cardiac computed tomography (CCT) offers superior resolution and demonstrates the morphology of the outpouching. Nuclear imaging techniques are less commonly used due to inherent low spatial resolution and availability of alternative modalities. Cardiovascular magnetic resonance imaging offers detailed visualization and tissue characterization. Early gadolinium enhancement images are particularly valuable for excluding thrombus within the aneurysm, while late gadolinium enhancement (LGE) highlights underlying issues such as transmural infarcts or infiltrative diseases. Aneurysms typically display LGE within the wall, indicating scarred and fibrotic myocardium. Conversely, the LGE of the pericardium above suggests a pseudoaneurysm, possibly due to chemical irritation, inflammation, and neovascularization during the acute phase of myocardial rupture. Despite the above imaging modalities, differentiating between aneurysms and pseudoaneurysms remains challenging. Novel techniques such as late enhancement dual-energy computed tomography iodine mapping and three-dimensional printing from CCT images have been reported to aid in evaluating left ventricular outpouchings and guiding surgery. It is important to identify LVAs promptly and accurately, as they can lead to left ventricular free wall rupture, which accounts for approximately 5%-24% of all in-hospital deaths related to MI [5]. Improving the classification and management of patients with these complications is essential to reduce the high mortality associated with them. These imaging modalities can distinguish various consistencies. For instance, a simple pericardial effusion would usually exhibit a density similar to that of water on CT scans (ranging from -20 to 20 Hounsfield units). In contrast, hemopericardium would typically display a significantly higher density on CT (exceeding 30 to 45 Hounsfield units) [6]. In our case, this distinction was evident as the mean density of the pericardial effusion measured 48.3 Hounsfield units, indicating the presence of hemopericardium, and substantiated the rupture of the aneurysm.

Pseudoaneurysms occur when the ventricular free wall ruptures but is contained by the surrounding adherent pericardium. In contrast, true aneurysms are characterized by areas of thinned myocardium that are dyskinetic and affect the entire thickness of the wall. They typically have a neck narrower than the diameter of the aneurysm and are frequently found in the posterior and lateral wall segments in contrast to true aneurysms, which are more often seen in the anterior wall and apex and have a wide neck. It is crucial to note that pseudoaneurysms pose a greater risk of rupture (30%-45%) and mortality (23%-48%) [7,8]. Several risk factors have been identified that increase the likelihood of LVA rupture, including age over 70 years, female gender, a first-time infarction, and a history of prior MI with an anterior location or a transmural infarction [2]. Both LVAs and pseudoaneurysms can lead to various complications, which may include thromboembolization, heart failure, ventricular arrhythmias, pericardial effusion with tamponade, and left ventricular rupture. Surgical intervention is a viable approach for managing these complications. In the case of LV pseudoaneurysms, once the diagnosis is confirmed, urgent surgical intervention is necessary as the risk of rupture outweighs the risk of surgery. In situations involving true aneurysms where surgery is not pursued or considered unsuitable, conservative management options include implementing guideline-directed medical therapy to address associated heart failure. Additionally, the possibility of percutaneous repair methods should be considered.

Recent advancements in the field of cardiology have led to a notable reduction in the occurrence of mechanical complications following an MI. In cases where these subsequent issues arise, their potential for morbidity and mortality is significant, often necessitating aggressive medical intervention [9]. The crucial step in managing patients with LVAs is conducting a thorough diagnostic workup that can effectively differentiate between an aneurysm and a pseudoaneurysm, as this distinction is pivotal in guiding the appropriate treatment approach [10].

Although LVAs are the most common mechanical complications following an MI, instances of contained aneurysm rupture leading to hemopericardium are infrequent and scarcely reported [11-14]. As physicians, we should be aware of ventricular and free wall rupture as uncommon but critical complications of MI, especially in cases with delayed presentations. Imaging should be pursued promptly when post-MI patients experience hemodynamic instability. Echocardiography serves as a valuable diagnostic tool for identifying these complications with minimal risk to the patient. In cases with pericardial effusion, contrast-enhanced echocardiography can assist in determining the etiology [15]. However, optimal strategies regarding the timing of intervention and the choice between surgical and percutaneous approaches remain uncertain and an area requiring further research.

## Conclusions

A contained rupture of LVAs causing hemopericardium is a rare occurrence, especially in cases of MI that present late. However, it is crucial to keep this possibility in mind when dealing with such cases. Prompt utilization of imaging techniques such as echocardiography is vital for an accurate diagnosis. When addressing the situation, determining the optimal timing for intervention, and choosing between surgical and percutaneous approaches remain areas that necessitate additional research to establish clearer guidelines and protocols.
